# Haematogenous abdominal wall metastasis of differentiated, alpha-fetoprotein-negative hepatocellular carcinoma after previous antiandrogen therapy within a site of lipoma manifestation since childhood

**DOI:** 10.1186/1477-7819-10-98

**Published:** 2012-05-30

**Authors:** L Zachau, C Zeckey, J Schlue, J Sander, C Meyer-Heithuis, M Winkler, J Klempnauer, H Schrem

**Affiliations:** 1 General, Visceral und Transplantation Surgery, Hanover Medical School, Carl-Neuberg-Str. 1, 30625, Hannover, Germany; 2 Trauma Department, Hanover Medical School, Carl-Neuberg-Str. 1, 30625, Hannover, Germany; 3 Institute of Pathology, Hanover Medical School, Carl-Neuberg-Str. 1, 30625, Hannover, Germany; 4 Department of Gastroenterology, Hepatology and Endocrinology, Hanover Medical School, Carl-Neuberg-Str. 1, 30625, Hannover, Germany

**Keywords:** Hepatocellular carcinoma, Cutaneous metastasis, Antiandrogen therapy

## Abstract

**Background:**

Cases with subcutaneous metastasis of differentiated hepatocellular carcinoma to the abdominal wall without prior seeding as a consequence of local interventions with a negative or normal alpha-fetoprotein level in the serum are extremely rare.

**Case report:**

This is the first report of a case with AFP-negative, differentiated hepatocellular carcinoma metastasis to the abdominal wall within a pre-existing subcutaneous lipoma since childhood after antiandrogen therapy with leuprorelin and buserelin acetate for prostate cancer without seeding.

**Methods:**

Clinical features including histology, immunohistochemistry, clinical course and surgical approach are presented.

**Results:**

Histological examination revealed a hepatocellular carcinoma with a trabecular and pseudoglandular growth pattern with moderately atypical hepatocytes with multifocal bile formation within a lipoma. The postoperative course of abdominal wall reconstruction with a monocryl-prolene mesh and a local flap after potentially curative resection was uncomplicated.

**Discussion and conclusion:**

It may be that previous antiandrogen treatment for prostate carcinoma contributed to the fact that our patient developed alpha-fetoprotein-negative and androgen receptor-negative subcutaneous abdominal wall metastasis within a pre-existing lipoma since childhood.

## Background

Hepatocellular carcinoma (HCC) remains one of the most common malignant diseases and the incidence is increasing worldwide [1-5]. Today, a multidisciplinary approach including surgical, chemotherapeutic and interventional therapeutic modalities is regarded as a prerequisite to enable optimal results. Distant metastases are common, the most frequent sites include the lungs, bones, lymph nodes and adrenal glands but extrahepatic metastasis to the abdominal wall is comparatively very rare [6-10].

Cases with subcutaneous differentiated metastasis of hepatocellular carcinoma with a negative or normal alpha-fetoprotein (AFP) level in the serum are extremely rare (see Table 1) [11-36].

**Table 1:** **Non-iatrogenic skin metastasis from hepatocellular carcinoma described in the literature the last 10 years**.

**Reference**	**N**	**Sex**	**Age**	**Localisation**	**Seeding**	**AFP ng/ml**	**Appearance**	**Hepatitis**
Jegou *et al*. (2004) [11]	1	M	55	left frontal	no	n.a.	painless nodule	n.a.
De Agustin *et al*. (2007) [12]	1	F	65	face	no	n.a.	n.a.	n.a.
Hsieh *et al*. (2007) [13]	1	M	46	left skull	no	71 ng/ml	soft mass	B
Royer *et al*. (2008) [14]	1	M	74	nose	no	n.a.	pearly purple nodule	n.a.
Magana and Gomez *et al*. (2009) [15]	1	M	41	right cheek	no	n.a	red papule	C
Lazaro *et al*. (2009) [16]	1	M	61	nasal lesion	no	n.a.	excoriated palpule	n.a.
Fukushima *et al*. (2010) [17]	1	M	58	left skull	no	n.a	n.a	n.a
Lee *et al*. (2010) [18]	1	M	49	tip of nose	no	n.a.	dark spot	C
Isa *et al*. (2011) [19]	1	M	81	left nasal alae	no	normal	multilobulated mass	negative
Ackerman *et al*. (2001) [20]	1	M	52	left scapula	no	10000 ng/ml	hemangio-sarcoma	B,C
Braune *et al*. (2001) [21]	1	M	76	right chest	yes	n.a.	subcutaneous tumor	n.a.
Al-Mashat FM (2004) [22]	1	M	60	chest wall	n.a.	n.a.	n.a.	n.a.
Amador *et al*. (2007) [23]	1	M	80	shoulder	no	n.a.	abscess-like	B
Terada *et al*. (2010) [24]	1	M	86	right chest	no	n.a	cutaneous mass	C
Suzuki *et al*. (2003) [25]	1	M	66	abdomen	n.a.	n.a.	subcutaneous nodule	C
Lee *et al*. (2004) [26]	1	M	68	right upper abdomen	yes	normal	red nodule	B
Hyun *et al*. (2006) [27]	1	M	56	left abdomen	no	308 ng/ml	n.a.	B
Nggada *et al*. (2006) [28]	1	M	53	chest, back abdomen,	no	negative	nodules	B
Martinez Ramos *et al*. (2007) [29]	1	W	70	subxyphoid region	yes	324000 ng/ml	violaceous cutaneous mass	C
Rowe *et al*. (2007) [30]	1	W	72	abdominal wall	yes	n.a.	nodule	no
Özdil *et al*. (2009) [31]	1	M	76	right abdomen	yes	1860 ng/ml	subcutaneous tumor	B
Chen *et al*. (2011) [32]	1	F	69	abdominal wall	yes	275-1217 ng/ml	n.a	C
Fang *et al*. (2001) [33]	1	M	49	back, hands, feet	no	n.a.	reddish-blue papules	n.a.
Kanitakis *et al*. (2003) [34]	1	F	50	right arm	no	increased	n.a.	n.a.
Nagaoka *et al*. (2004) [35]	1	F	77	right waist	yes	8 ng/ml	reddish papules	n.a.
Masannat *et al*. (2007) [36]	1	M	63	left calf	no	5 ng/ml	like soft-tissue sarcoma	n.a.

(AFP, alpha-fetoprotein; n.a.: not applicable).

Most cases with metastasis to the abdominal wall are described after percutaneous interventions including fine needle aspiration biopsy (FNAB), cytology, radiofrequency ablation or percutaneous ethanol injection (PEI). In the current literature during the last 10 years, only few cases have been reported with abdominal wall metastasis without prior seeding as a consequence of local interventions (see Tables 1 and 2).

**Table 2:** **Study with 21 patients with 24 non-iatrogenic subcutaneous HCC metastases from 1 January 1998 to 31 December 2005**[53]

	**Head**	**Chest**	**Back**	**Abdominal wall**	**Arms and legs**
Huang Y-J *et al*. (2008) [53]	8	6	4	5	1

Long-term study on non-iatrogenic subcutaneous HCC metastases.

We present a case of a 75-year-old man with singular abdominal wall metastasis of differentiated, AFP-negative hepatocellular carcinoma without a history of prior seeding and without prior surgery to the abdominal wall. This is the first report of a case with hepatocellular carcinoma metastasis to the abdominal wall within a pre-existing subcutaneous lipoma since childhood and after antiandrogen therapy for prostate cancer since 2005.

This article provides a surgical approach, including abdominal wall reconstruction, and a current review of the literature as cited in PubMed.

## Case presentation

### Preoperative diagnostics and medical history

Our patient was referred to our center in March 2010 for diagnostic evaluation of a growing liver mass in liver segment II (3.9 × 2.6 × 3.0 cm). He was in good general condition with an obese nutritional status (Body Mass Index 30.4 kg/m²) and an impression of fatty liver parenchyma in ultrasound and magnetic resonance imaging. He was reported to drink two glasses of whisky per day. The previous history included multiple subcutaneous lipomas within the abdominal wall since childhood and prostate carcinoma, which was diagnosed in 2005 and removed by prostatectomy (pT2, pN0, MO, G3) in the same year, followed by antiandrogen therapy with leuprorelin and buserelin acetate until August 2008. The multiple lipomas showed a marked progression in size under antiandrogen therapy from August 2007 until August 2008. Final diagnostic evaluation, including ultrasound-guided FNAB of the liver mass and magnetic resonance imaging, resulted in the diagnosis of a multilocular, well-differentiated and AFP-negative hepatocellular carcinoma in liver segments II/III, VI, and VIII in April 2010. AFP levels stayed within normal range at all times during further follow-up. Viral hepatitis or other risk factors for hepatocellular carcinoma like hemochromatosis, liver fibrosis or liver cirrhosis could be ruled out. After interdisciplinary tumor board consultation (including surgeons, oncologists, radiologists, radio-oncologists and hepatologists) transarterial chemoembolisation (TACE) was performed from July 2010 to January 2011 every two months leading to a complete remission.

**Figure 1:**
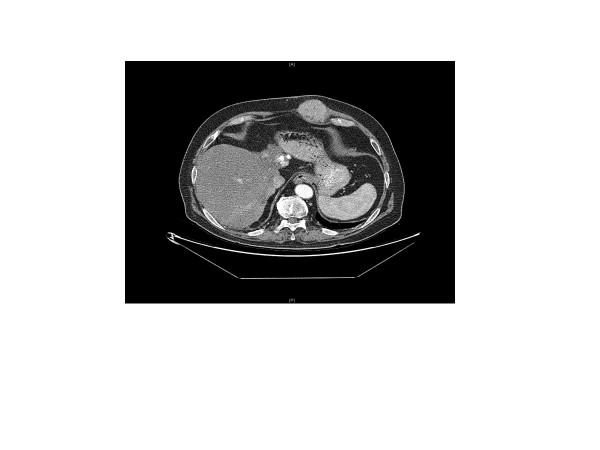
Computed tomography scan showing a tumor with a diameter of 7 cm in the left upper abdominal wall.

**Figure 2:**
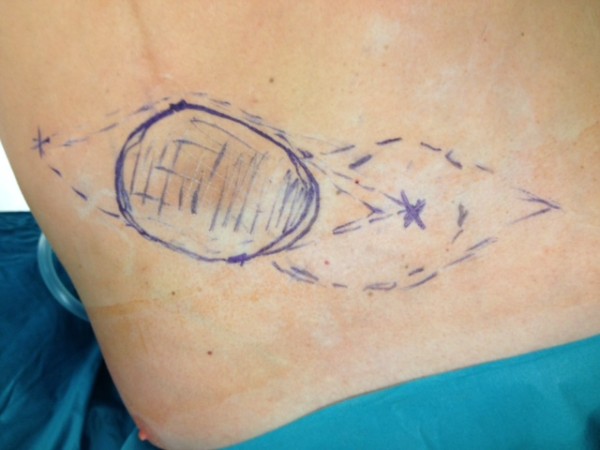
Preoperative marking lines for the flap plastic.

Consultation with our surgical service was initiated in November 2011 due to a hard palpable subcutaneous mass with a diameter of approximately 7 cm that had developed recently in the left upper abdominal wall, exactly where a soft subcutaneous lipoma manifestation has been, in the consciousness of the patient, since his childhood (see Figures 1 and 2). The patient assured us that FNAB had not been performed in the region of the pre-existing multiple lipomas in the left upper abdominal wall. This was consistent with the documentation of previous diagnostic work-up. Our patient was concerned about the marked change in size and consistency of the tumor in the left abdominal wall with concomitant darkening of the skin directly adjacent to the tumor (see Figure 2). Due to the patient’s medical history, the newly occurred subcutaneous tumorous mass in the left abdominal wall had to be suspected to be malignant. After ultrasound evaluation of the subcutaneous mass, a restaging computed tomography was initiated that demonstrated a tumor with a diameter of 7 cm within the left upper abdominal wall with no signs of lymph node metastasis, lung metastasis or any current hepatic tumor (see Figure 1). After interdisciplinary tumor board consultation (including surgeons, oncologists, radiologists, radio-oncologists and hepatologists) it was decided to recommend tumor resection and abdominal wall reconstruction.

### Surgical therapy

**Figure 3:**
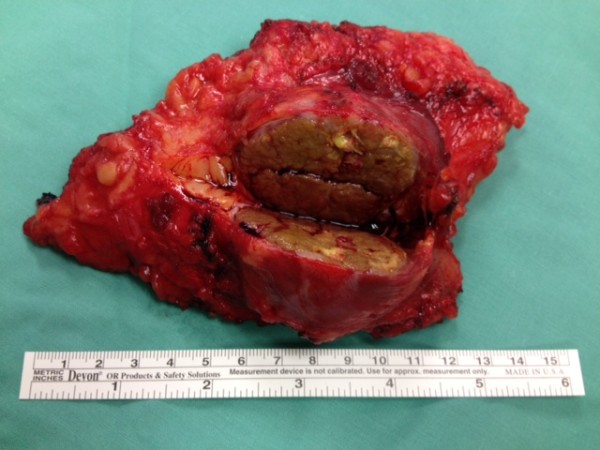
**Shown is the resected abdominal wall metastasis.** The resection margins were macroscopically and microsopically tumor-free.

**Figure 4:**
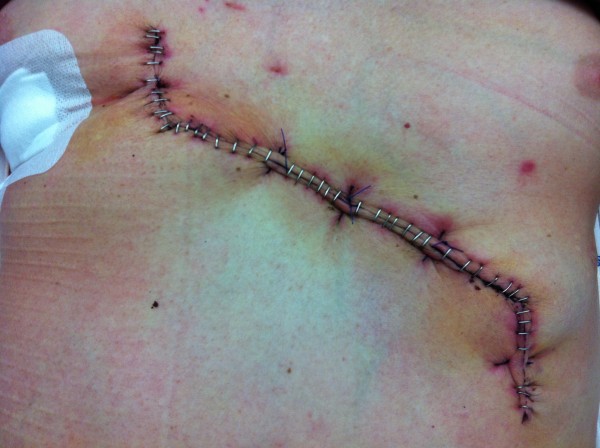
Abdominal wall six days after tumor resection with subcutaneous flap plastic and staple sutures.

We removed the tumor by resection of the skin with surrounding subcutaneous tissue *en bloc* with the surrounding upper left rectus abdominis muscle including the fascia. The resulting abdominal wall defect was too large for primary closure or component separation. We therefore decided to reconstruct the large defect in the abdominal wall fascia with a synthetic, partially resorbable monocryl-prolene mesh that we inserted into the defect of the fascia with a running monocryl-prolene suture without opening the peritoneal cavity. Fortunately, the peritoneum with the subfascial fat tissue layer could be easily separated from the tumor without risking incomplete resection. It was macroscopically evident that the peritoneum was not infiltrated by the tumor. In order to enable a tension-free primary wound and skin closure, we covered the mesh with a local flap and installed two Redon drains (size 12 Charrière) subcutaneously with suction for a total of five days (see Figures 2, 3 and 4).

### Postoperative clinical course and histopathology

The clinical course after resection was uneventful. There was primary wound healing without any signs of infection or hematoma. Thus, the inserted drains could be removed after five days. Laboratory analysis evidenced slightly elevated infectious parameters during the first postoperative days, probably due to the surgical stimulus; however, these declined and were within normal range after hospital discharge. The mobilization was uneventful as well as oral food intake and intestinal motility.

**Figure 5:**
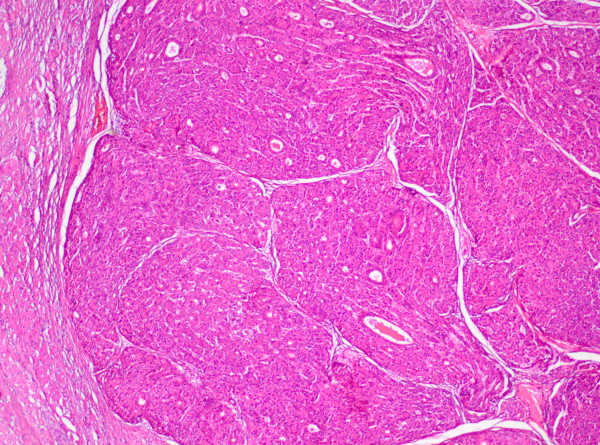
Hepatocellular carcinoma with trabecular and pseudoglandular growth pattern (H&E stain, original magnification 20x).

**Figure 6:**
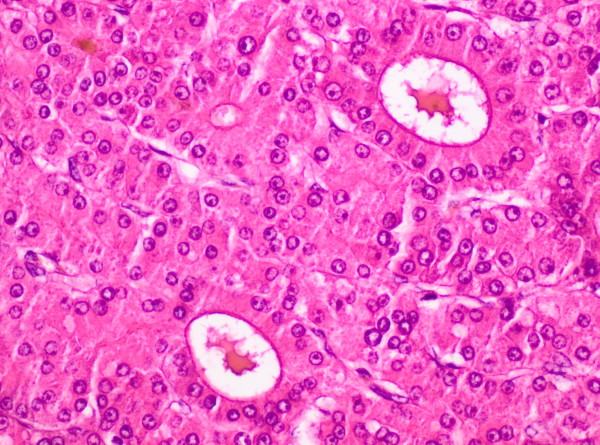
Hepatocellular carcinoma with moderately atypical hepatocytes with multifocal bile formation (H&E stain, original magnification 200x).

**Figure 7:**
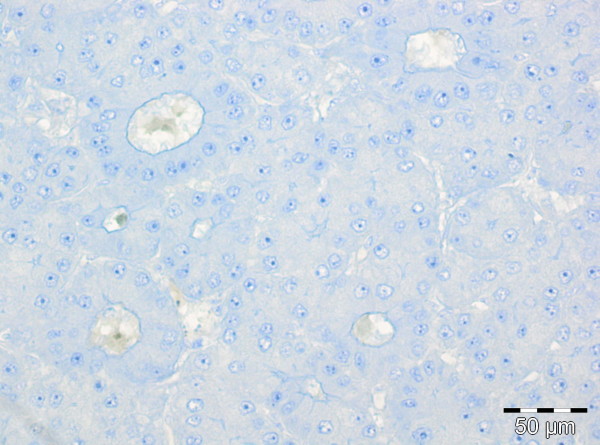
Immunohistochemical staining for androgen receptor negative in the tumor cell nuclei (original magnification 200x).

**Figure 8:**
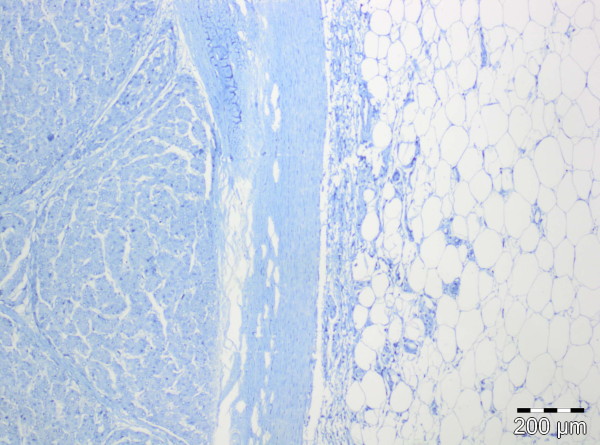
Immunohistochemical staining for estrogen receptor: negative in the HCC (left) and lipomatous tissue (right) (original magnification 40x).

**Figure 9:**
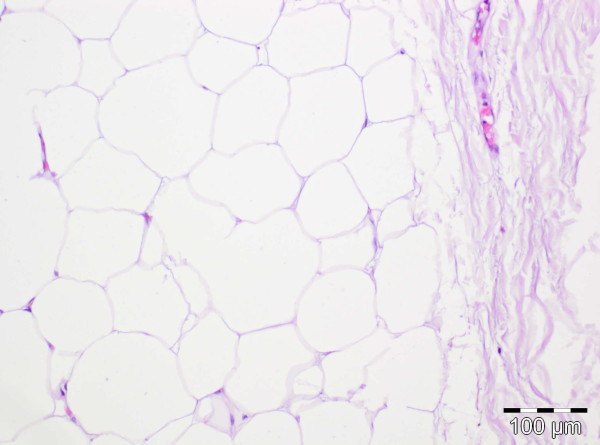
Detail from the lipoma: mature adipocytes with no atypia and a delicate fibrous tumor capsule (right, H&E stain) (original magnification 100x).

Final pathological workup showed a hepatocellular carcinoma with a trabecular and pseudoglandular growth pattern (see Figure 5) with moderately atypical hepatocytes (G2) and multifocal bile formation without steatosis (see Figure 6). Immunohistochemistry was performed according to standard procedures using monoclonal antibodies for human androgen receptor (mouse clone AR441, Dako, Hamburg, Germany) as well as estrogen and progesterone receptor (rabbit SP1, and mouse PgR636, respectively, Dako) and polyclonal for AFP (rabbit, Dako). Multiple tissue blocks from different sites of the HCC showed no expression of the androgen receptor (see Figure 7). Furthermore, there was no detectable expression of AFP, estrogen or progesterone receptor. By gross examination, a subcutaneous lipomatous nodule of about 3 × 2 × 1.5 cm adjacent to the HCC could be identified. The histological findings were well compatible with the diagnosis of a lipoma, the sex hormone receptor status of the adipocytes was negative (Figures 8, 9).

## Discussion

Abdominal wall metastasis of primary hepatocellular carcinoma is generally a comparatively rare entity (for a summary of previously published cases, see Tables 1 and 2). Mostly described as the consequence of tumor seeding after percutaneous interventions, the metastatic potential of hepatocellular carcinoma without prior seeding has so far not been described in detail. The pathophysiological process of abdominal wall metastasis without prior seeding is poorly understood. In cases with liver cirrhosis, portal venous backflow mechanisms via collaterals in the abdominal wall may play a hypothetic role for the development of abdominal wall metastasis. Our patient had no cirrhosis and no significant portal venous collaterals in the abdominal wall.

Our patient underwent a previous prolonged period of antiandrogen therapy with leuprorelin and buserelin acetate with the intention to treat his prostate carcinoma until August 2008. It well may be that this treatment significantly contributed to the fact that our patient developed a differentiated, AFP-negative abdominal wall metastasis instead of undifferentiated HCC. Of note in this context is the histomorphologically mature phenotype of the HCC metastasis, the neoplastic hepatocytes showing no steatosis and no expression of the androgen receptor.

It is interesting to note that the abdominal wall lipoma demonstrated an accelerated growth during antiandrogen therapy. This may have contributed to haematogenous spread of HCC to the abdominal wall. Interestingly, numerous lines of evidence point to a role of androgens in the development of hepatocellular carcinoma [37-42].

It could be demonstrated that the majority of hepatocellular carcinoma cells express androgen receptors in higher levels as compared to normal hepatocytes [37-42]. Taken together epidemiologic, clinical, biologic and experimental studies have provided a strong rationale for antiandrogen therapy in hepatocellular carcinoma (HCC). Epidemiological studies demonstrated that the sex ratio for HCC arising in cirrhotic patients is eleven men for one woman, whereas the sex ratio in cirrhotic patients without HCC is less than two men for one woman [43]. Animal studies demonstrated that carcinogens induce HCC more easily in males than females. Matsumoto *et al*. could demonstrate that transforming growth factor alpha (TGF-alpha)-related hepatocarcinogenesis and hepatocyte proliferation are increased by androgenic stimulation in TGF-alpha transgenic mice [44]. Several clinical studies have reported occurrence of HCC after administration of androgen therapy (for example, with testosterone) in male subjects who suffer from aplastic anemia or infertility [45-47]. Interestingly, stopping androgen therapy usually led to regression of the tumor [38].

Androgen receptors have been demonstrated in HCC in 74 % of males and 37 % of females, and their presence is associated with a more rapid progression of disease [40]. Arguments against our speculation that previous antiandrogen therapy may have caused the abdominal wall metastasis in our patient to be differentiated and AFP-negative may be derived from more recent data and the fact that immunhistochemical staining was negative in the tumor cell nuclei of our patient (see Figure 7). Antiandrogens (for example. leuprorelin) have been used in prospective randomized multicenter trials to treat hepatocellular carcinoma [38,39]. One study with leuprorelin and flutamide demonstrated no benefit in survival for male patients with HCC treated with antiandrogens [39]. Another study showed a lack of efficacy of androgen treatment in unresectable HCC [38].

More recent studies with Cre-Lox conditional knockout mice model who lack androgen receptor (AR) expression demonstrated that the androgen receptor, but not androgens, may be a potential new and better therapeutic target for the treatment of HCC [48]. A new animal study found that hepatic androgen receptor promotes hepatitis B virus (HBV)-induced hepatocarcinogenesis in HBV transgenic mice that lack AR only in the liver hepatocytes (HBV-L-AR(−/y). HBV-L-AR(−/y) mice that received a low dose of the carcinogen N'-N'-diethylnitrosamine have a lower incidence of HCC and present with smaller tumor sizes, fewer foci formations, and less alpha-fetoprotein HCC marker than do their wild-type HBV-AR(+/y) littermates [49]. Taken together it appears that the androgen receptor in HCC may be more relevant for the biology of the tumor than the androgens or antiandrogen treatment.

Due to the low incidence of subcutaneous abdominal wall metastasis of HCC, therapeutic algorithms are widely missing. In the current literature, surgical resection is the favored therapeutic option [13,23,32]. However, surgical resection might be challenging depending on tumor size, invasiveness and localization. Immediate definitive closure of the wound is the preferred method. In cases with distinct wound distension, local inflammation or wound infect, temporary abdominal wall closure should be considered [50]. Meanwhile, abdominal wall reconstruction is necessary due to the resulting abdominal wall defect. Described methods include prosthetics or autologous reconstruction such as abdominal components separation or local flaps. Generally, deeper and larger wound defects that include the myofascia may require a synthetic, partially resorbable monocryl-prolene mesh to replace the resected myofascia that may have to be covered by a free tissue or a distant flap in order to reduce wound tension [51]. The local flap used in this case to cover the implanted partially resorbable monocryl-prolene mesh for abdominal wall reconstruction is a well-established procedure and proved successful during follow-up in this case [52]. In our opinion, resection of hepatocellular carcinoma metastases to the abdominal wall with peritoneal infiltration may require a modified approach for abdominal wall reconstruction, for example, with the use of a dual-layer mesh that is able to reduce postoperative adhesions between the intestine and the mesh within the intraperitoneal cavity. Fortunately in this case, opening of the peritoneal cavity could be avoided.

Huan *et al*. reported 21 patients with a subcutaneous HCC metastasis without seeding who were treated by radiotherapy (see Table 2) [53].

Single cases are not discussed in detail, so comparison with our work or the reports of other cases is barely possible. There is a substantial lack of evidence for adjuvant chemotherapeutic approaches after potentially curative resection of abdominal wall metastasis. In order to establish therapeutic algorithms for cutaneous metastasis of hepatocellular carcinoma, the initiation of large multicenter studies would be desirable in order to be able to create scientific evidence [54,55]. Due to the rarity of cutaneous metastasis of hepatocellular carcinoma, this is a major and potentially unsolvable challenge.

## Conclusions

We believe that in our case the abdominal wall metastasis is a consequence of systemic arterial tumor spread to a pre-existing lipoma since childhood via its blood supply. To our knowledge, this is the first report of such a case. Our case highlights that suspicion of abdominal wall metastasis of hepatocellular carcinoma is also justified in patients with pre-existing, long-standing palpable abdominal lipoma since childhood, especially when apparent growth of these nodules is being observed. We recommend surgical removal and abdominal wall reconstruction in such cases in palliative intention to avoid tumor necrosis at the abdominal wall and/or infiltration of the small or large bowel followed by potential percutaneous bowel perforation. Cases with a singular hepatocellular metastasis to the abdominal wall with otherwise complete surgical resection of the primary tumor may benefit from resection with potential improvement of survival.

## Consent

Written informed consent was obtained from the patient for publication of this case report and any accompanying images. A copy of the written consent is available for review by the Editor-in-Chief of this journal.

## Abbreviations

AFP: alpha-feto protein; AR: androgen receptor; FNAB: fine needle aspiration biopsy; H&E: hematoxylin and eosin; HBV: hepatitis B virus; HCC: hepatocellular carcinoma; n.a: not applicable; PEI: percutaneous ethanol injection; TACE: transarterial chemoembolisation; TGF-alpha: transforming growth factor alpha.

## Competing interests

The authors declare they have no competing interests. The authors have no conflict of interest to disclose which may have biased their work. The authors declare that they did not receive any funding for this work.

## Authors’ contributions

LZ operated on the patient, collected the majority of relevant clinical data and drafted the manuscript. CZ was involved in the collection of data and helped significantly with the writing of the manuscript. SJ carried out the histological examination of the resected specimen, including immunohistochemistry, and helped with the writing of the manuscript. JTS researched the literature for this case and contributed significantly with tables and relevant intellectual comments on the manuscript. CMH helped significantly with the writing of the manuscript and contributed an important interdisciplinary viewpoint that helped substantially improve the intellectual content of the manuscript. MW participated in the design of the report and helped to improve the intellectual content of the manuscript. JK provided relevant surgical experience and advice and helped to improve the intellectual content of the manuscript. HS had the idea to write this case report, participated significantly in its design and coordination and helped to improve the intellectual content of the manuscript. All authors read and approved the final manuscript.

## Authors’ information

All authors of this article are clinically highly experienced physicians who are regularly involved with interdisciplinary treatment decisions for patients with hepatocellular carcinoma (HCC), encompassing up-to-date interventional, chemotherapeutic, and surgical approaches, including liver resection and liver transplantation, in one of the largest academic treatment centres for HCC in Germany.
